# Investigating complex I deficiency in Purkinje cells and synapses in patients with mitochondrial disease

**DOI:** 10.1111/nan.12282

**Published:** 2015-09-30

**Authors:** Alexia Chrysostomou, John P. Grady, Alex Laude, Robert W. Taylor, Doug M. Turnbull, Nichola Z. Lax

**Affiliations:** ^1^ Wellcome Trust Centre for Mitochondrial ResearchInstitute of NeuroscienceNewcastle UniversityNewcastle upon TyneUK; ^2^ Bio‐imaging UnitNewcastle UniversityNewcastle upon TyneUK

**Keywords:** ataxia, mitochondrial disease, mitochondrial DNA, Purkinje cells, respiratory chain deficiency, synapses

## Abstract

**Aims:**

Cerebellar ataxia is common in patients with mitochondrial disease, and despite previous neuropathological investigations demonstrating vulnerability of the olivocerebellar pathway in patients with mitochondrial disease, the exact neurodegenerative mechanisms are still not clear. We use quantitative quadruple immunofluorescence to enable precise quantification of mitochondrial respiratory chain protein expression in Purkinje cell bodies and their synaptic terminals in the dentate nucleus.

**Methods:**

We investigated NADH dehydrogenase [ubiquinone] 1 alpha subcomplex subunit 13 protein expression in 12 clinically and genetically defined patients with mitochondrial disease and ataxia and 10 age‐matched controls. Molecular genetic analysis was performed to determine heteroplasmy levels of mutated mitochondrial DNA in Purkinje cell bodies and inhibitory synapses.

**Results:**

Our data reveal that complex I deficiency is present in both Purkinje cell bodies and their inhibitory synapses which surround dentate nucleus neurons. Inhibitory synapses are fewer and enlarged in patients which could represent a compensatory mechanism. Mitochondrial DNA heteroplasmy demonstrated similarly high levels of mutated mitochondrial DNA in cell bodies and synapses.

**Conclusions:**

This is the first study to use a validated quantitative immunofluorescence technique to determine complex I expression in neurons and presynaptic terminals, evaluating the distribution of respiratory chain deficiencies and assessing the degree of morphological abnormalities affecting synapses. Respiratory chain deficiencies detected in Purkinje cell bodies and their synapses and structural synaptic changes are likely to contribute to altered cerebellar circuitry and progression of ataxia.

## Introduction

Mitochondrial diseases are a clinically heterogeneous group of genetic disorders underpinned by mitochondrial respiratory chain dysfunction. The mitochondrial respiratory chain is responsible for the generation of ATP via oxidative phosphorylation (OXPHOS) and is under dual genetic control [1]. Mitochondrial disease can be caused by either mutations in the mitochondrial DNA (mtDNA) (point mutations or rearrangements) or mutations in nuclear DNA (nDNA)‐encoded proteins involved in mitochondrial homeostasis [2].

Neurons are exquisitely sensitive to fluctuations in mitochondrial function and ATP generation. They possess a highly specialized architecture with long processes extending from the cell body to the distal synapses projecting onto other neuronal cells. Energy supply to these distal regions is achieved via motor protein‐assisted mitochondrial transport along the axons [3]. Sites with increased ATP demand have a higher density of mitochondria [4], and synapses are among the most energetically active parts of a neuron. In the synapse, mitochondria regulate numerous processes including synaptic vesicle exocytosis and recycling, and calcium buffering [5, 6, 7]. It is, therefore, not surprising that neurological deficits are widespread in patients with mitochondrial disease, and symptoms may be manifold including seizures, cognitive decline and cerebellar ataxia [8]. Recent studies indicate that approximately 70% of patients recruited to the UK MRC Mitochondrial Disease Patient Cohort are affected by cerebellar ataxia which is debilitating and progressive. The cerebellum is particularly vulnerable to mitochondrial dysfunction, and neuropathological investigation of patients often reveals cerebellar atrophy, Purkinje cell loss and mitochondrial respiratory chain deficiencies of complex I in remaining cells [9, 10, 11, 12, 13, 14].

Evidence of synaptic pathology has been documented in post‐mortem brain tissue from patients with mitochondrial disease, including decreased synaptophysin immunoreactivity and swollen (when present) calbindin‐positive endings to the dentate nucleus neurons [9, 10]. Genetic manipulation of proteins involved in mitochondrial fission and fusion in cultured neurons [15] and mice [16] has revealed mitochondrial retraction towards the cell body accompanied by striking alterations in synaptic integrity. Observed changes include a reduction in dendritic spine density, decreased synaptic formation and delayed transmitter release frequency [15, 16]. Artificial inhibition of mitochondrial respiratory chain function *in vivo* and *in vitro* revealed the importance of ATP levels in controlling synaptic energetic state and in maintaining synaptic integrity and functionality [17, 18]. Recent work has recognized synaptic dysfunction and degeneration in response to mitochondrial dysfunction as an early feature in neurodegenerative disease [19, 20].

In this study, we aimed to clarify the degree of deficiency and changes in synaptic structures in the cerebellum of patients with adult‐onset mitochondrial disease. The cerebellum is a clinically relevant and well‐defined structure in which detailed neuropathological studies have been performed previously in patients with mitochondrial disease; however, there have been no in depth studies investigating the presence of respiratory chain defects or structural changes affecting the synapse. We quantified respiratory chain deficiency in Purkinje cells and their gamma aminobutyric acid (GABA)‐ergic synapses contacting neurons in the dentate nucleus, evaluating presynaptic terminal structural characteristics.

## Materials and methods

### Brain tissue samples

Formalin‐fixed paraffin‐embedded (FFPE) and frozen cerebellum tissue from 12 adult patients with clinically and genetically defined mtDNA disease was obtained from the Newcastle Brain Tissue Resource (NBTR), while cerebellum tissue from 10 aged‐matched controls was obtained from the NBTR and the MRC Sudden Death Brain and Tissue Bank, Edinburgh (Table S1). There are no statistically significant differences in age (Mann–Whitney *U* test, *P* value = 0.3390) and post‐mortem interval (PMI; Mann‐Whitney *U* test, *P* value = 0.6209) in patients and controls selected. Patient details are summarized in Table 1 [9, 21, 22, 23]. Newcastle and North Tyneside Local Research Ethics Committee (LREC2002/205) approved this study, and full consent for brain tissue retention and research was obtained.

**Table 1:** Clinical summary of patients

	Pt1	Pt2	Pt3	Pt4	Pt5	Pt6	Pt7	Pt8	Pt9	Pt10	Pt11	Pt12
Age	36	60	30	45	20	57	42	58	59	79	55	55
Gender	Female	Female	Male	Male	Female	Female	Female	Male	Male	Male	Male	Male
Genotype	m.3243A > G	m.3243A > G	m.3243A > G	m.3243A > G	m.3243A > G	m.3243A > G	m.8344A > G	m.8344A > G	*POLG* (p.Gly848Ser and p.Ser1104Cys)	*POLG* (p.Thr251Ile/p.Pro587Leu; p.Ala467Thr )	*POLG* (p.Trp748Ser and p.Arg1096Cys)	m.14709T > C
Disease duration (y)	15	33	11	ND	10	32	37	23	37	23	50	21
Fixation length (weeks)	1	6	10	8	6	50	8	6	4	8	7	17
Post‐mortem Interval (hours)	42	10	69	43	187	36	59	66	67	85	112	10
Percentage cell loss												
Purkinje cells (%)	59	70	0	ND	ND	59	67	8	65	45	ND	30
Dentate nucleus neurons (%)	56	0	20	ND	ND	56	75	48	8	31	ND	74
Neuropathology	Microinfarcts affecting the cerebellar cortex. Complex I and IV deficiency in ION, PCs and DNNs. Decreased synaptophysin immunoreactivity in DN. Vascular COX deficiency in brain parenchyma	Microinfarcts in cerebellar cortex. Complex I deficiency in remaining PCs and vascular COX deficiency	−	−	Microinfarcts in cerebellar cortex. Complex I deficiency in remaining PCs and complex I and IV deficiency in SNNs. COX deficiency and mineralization of blood vessels	Cerebellar lesions and mild (when existent) complex I and IV deficiency in remaining PCs and DNNs. Thinning of the endothelial cell layer	High levels of complex I and moderate levels of complex IV deficiency in surviving neurons of the olivo‐cerebellar pathway. Shrunken, eosinophilic neurons and decreased synaptophysin immunoreactivity. COX‐deficient vessels	Intact SN neuronal population and low levels of complex I deficiency	Cerebellar lesions and moderate complex I and IV deficiency in surviving neurons	−	−	Severe complex I and IV deficiencies in IO. Moderate complex I and mild complex IV deficiency in PCs and DNNs. Presence of ‘ghost’ synapses.
Ataxia (NMDAS rating)	3/5	3/5	1/5	ND	5/5	1/5	5/5	4/5	3/5	3/5	5/5	3/5
Epilepsy	+	+		+	+	+	+	+			+	
Publications	[9, 21, 23]	[9, 21, 23]	−	[9, 21, 23]	[9, 21, 22, 23]	[9, 21]	[9, 21, 22, 23]	[22, 23]	[9]	[23]	[23]	[9]

Clinicopathological details for patients with mitochondrial disease included in the study. Their genetic defect, disease duration, fixation length, post‐mortem interval, degree cell loss and NMDAS score for cerebellar ataxia are summarized. ND, not determined.

The patients with mitochondrial disease were clinically assessed using the validated Newcastle Mitochondrial Disease Adult Scale (NMDAS) [24] (Table 1) within 1 year of death. NMDAS scores for cerebellar ataxia confirmed cerebellar ataxia in all patients included in the study. Scaling varies from 0 to 5 where 0 is unaffected and 5 is wheelchair dependent.

### Two‐dimensional neuron counting and neuronal density calculation

Neuron densities for Purkinje cells and dentate nucleus neurons have been documented using a two‐dimensional counting protocol previously described [9]. In this study, quantification of Purkinje cell and dentate nucleus neuronal density was performed for Patients 3, 8 and 10 (Table 1) as they were not included in the previous study.

### Immunofluorescence to detect mitochondria and complex I in Purkinje cells and their synapses

Immunofluorescence was performed on 5 μm‐thick FFPE sections of cerebellar tissue mounted on SuperFrost slides (ThermoScientific, UK). Immunofluorescence was performed on positive controls (all antibodies), no‐primary‐antibody and no‐secondary‐antibody (NSA) controls for each of the four fluorophores to allow for background correction and cross‐reactivity checks respectively (Figure S1). Sections were deparaffinized and rehydrated by placing in a 60°C oven for 20 min, followed by immersion in Histoclear (National Diagnostics, Charlotte, NC, USA) and graded ethanol series (100% to 70%) to water. Antigen retrieval was performed using the 2100 retriever unit (Electron Microscopy Sciences^©^, Hatfield, PA, USA) which involved immersion of sections in 1 mmol EDTA (pH 8) and pressure cooking for 40 min. Sections were blocked in 1% normal goat serum (NGS) for 1 h at room temperature (RT) and incubated in primary antibodies at the optimal dilution overnight at 4°C. Mouse monoclonal primary antibodies used were directed against nDNA‐encoded respiratory chain complex subunits, NADH dehydrogenase [ubiquinone] 1 alpha subcomplex subunit 13 (anti‐NDUFA13; Abcam, UK, ab110240) and cytochrome c oxidase subunit 4 (anti‐COX4 (+4L2); Abcam, UK, ab110261), and a presynaptic protein synaptophysin (anti‐SY‐38; DAKO, UK, M0776). A rabbit polyclonal antibody was directed against Glutamic acid decarboxylase (anti‐GAD‐65/67; Sigma‐Aldrich, UK, G5163) (Table S2). Following incubation with the primary antibodies, sections were washed with 10 mM phosphate‐buffered saline (3 × 5 min) and subjected to a biotinylated secondary antibody incubation (Biotin‐XX goat anti‐mouse IgG1; Life Technologies, UK, A10519) for 30 min at RT. Quadruple immunofluorescent labelling involved secondary anti‐mouse antibodies conjugated with Alexa Fluor 488 and 546, anti‐rabbit conjugated with Alexa Fluor 405 antibody and streptavidin‐conjugated Alexa Fluor 647 antibody (Life Technologies, UK) (Table S2). The signal‐to‐noise ratio was increased by quenching the background signal with 3% Sudan Black for 10 min. Sections were then washed in distilled water and mounted in Prolong Gold (Life Technologies, UK).

### Confocal microscopy

Purkinje cells and synapses were imaged on x, y and z planes using an inverted point scanning confocal microscope (Nikon A1R, UK). Areas of interest (either dentate nucleus neurons or Purkinje cell bodies) were detected using an immersion oil ×60 objective with numerical aperture of 1.4. Twenty Purkinje cells and dentate nucleus neurons were randomly sampled per case using an electronic zoom of ×3 (× 180 magnification). Z‐stacking was performed according to the recommended microscope settings (pixel size: 0.14 μm; z‐step size: 0.17 μm; optical resolution: 0.13 μm and optical sectioning: 0.54 μm). Four lasers were employed (405 nm, 488 nm, 561 nm and 647 nm), each detecting the signal from a different fluorophore. Microscope and laser settings were maintained throughout image capture.

Laser stability and point spread function (PSF) measurements were achieved using FocalCheck™ Fluorescence Microscope Test slides (Molecular Probes™, Invitrogen, UK) and TetraSpeck™ Fluorescent Microsphere Standards (Invitrogen, UK) respectively.

### Image processing and three‐dimensional reconstruction of synapses

To investigate complex I (NDUFA13) and complex IV (COX4) protein expression in either Purkinje cell bodies or GABAergic synapses, three‐dimensional images were analysed using Volocity® 3D image analysis and deconvolution software (v.6.1.1, PerkinElmer, UK). Initially, all the images were cropped to single neurons; these were then deconvolved in three dimensions using measured PSFs.

Purkinje cells are the sole output of the cerebellar cortex and the only neurons to project inhibitory (GABAergic) synapses to deep cerebellar nuclei, such as the dentate nucleus [25]. Purkinje cells project their GABAergic axonal terminals onto dentate nucleus neurons, forming axosomatic inhibitory synapses. Different analysis protocols were developed depending on the cerebellar neuron (Purkinje cells *vs.* dentate nucleus neurons) and subneuronal compartment (cell bodies *vs.* GABAergic presynaptic terminals) of interest.

Glutamic acid decarboxylase 65/67 (GAD‐65/67) is uniformly and highly expressed in GABAergic neuronal cell bodies (GAD67) and inhibitory presynaptic terminals (GAD65) [26]. Synaptophysin is a component of presynaptic terminals and demonstrates variably strong puncta surrounding neuronal cell bodies. COX4 protein expression was strong, and the pattern of staining was uniform and punctate in both patients and controls in agreement with previous studies that rarely report its loss from the cerebellum [9] and therefore justifying its use as a mitochondrial mass marker. Complex I subunit NDUFA13 was selected as a marker for complex I deficiency as decreased NDUFA13 protein expression was previously detected in our patients [9], and because this antibody has a different isotype to the remaining three antibodies used, therefore avoiding antibody cross‐reactivity. Therefore, by using NDUFA13 in combination with COX4, we can determine the relative loss of this complex I subunit.

**Figure 1:**
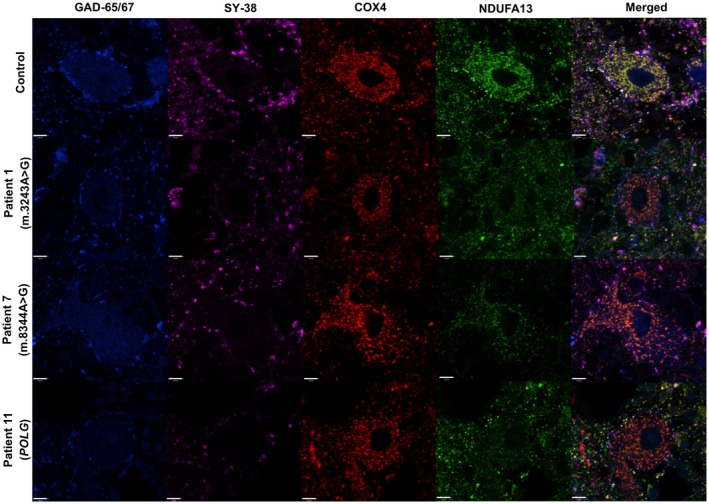
Detecting and quantifying mitochondrial protein expression in Purkinje cells. Quadruple immunofluorescence of cerebellar Purkinje cells against Glutamic acid decarboxylase (GAD‐65/67), synaptophysin (SY‐38), complex IV subunit 4 (COX4) and complex 1 alpha subcomplex subunit 13 (NDUFA13). GAD‐65/67 is used to detect GABAergic cell bodies and inhibitory synapses. Combined with SY‐ 38 indicates the convergence of inhibitory presynaptic terminals. COX4 is a mitochondrial mass marker, and NDUFA13 protein is used to detect complex I deficiency. Control Purkinje cells demonstrate co‐localization of COX4 and NDUFA13 protein, indicating intact complex I expression, while NDUFA13 protein is significantly reduced in patient Purkinje cells (complex I‐deficient). Purkinje cells in the three examples shown above (Patients 1, 8 and 11) exhibit variably decreased NDUFA13 protein relative to the amount of COX4 present. Scale bar: 7 μm.

**Figure 2:**
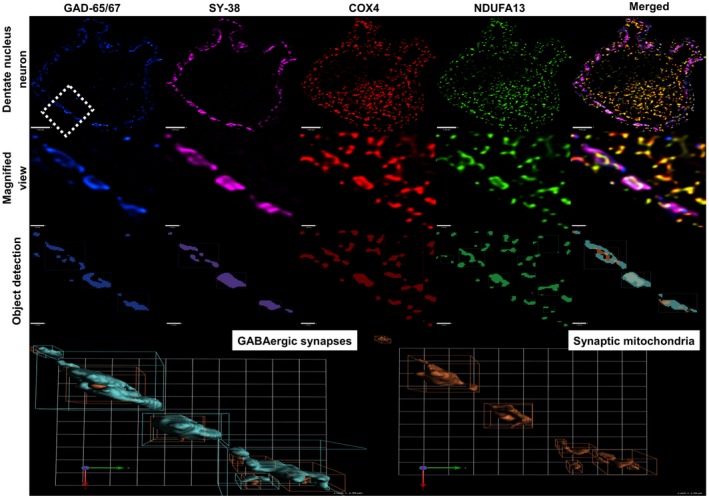
Detecting synaptic mitochondria and reconstructing GABAergic synapses. Development of the protocol for detecting and reconstructing GABAergic presynaptic terminals onto dentate nucleus neurons. Top panel: Confocal images of immunofluorescently stained control dentate nucleus neurons were used to identify inhibitory synapses. Scale bar: 7 μm. Magnified view: Areas around the periphery of neurons where GAD‐65/67 (blue) and synaptophysin (purple) puncta coexist are denotative for the presence of inhibitory synapses. COX4‐positive staining (red) indicates mitochondrial mass, and NDUFA13 presence (green) is used to detect respiratory chain protein deficiency. Scale bar: 1.5 μm. Object detection: Actual staining substituted by objects that represent each of the four markers used. Mitochondrial objects can then be compartmentalized and filtered so that only the ones that are within synapses are studied (far right). Bottom panel: Three‐dimensional reconstruction of inhibitory synapses on the same control neuron, and identification of synaptic mitochondria. Scale: 1 unit = 1.56 μm. Protocol development is achieved using the Volocity® software (v.6.3.1, PerkinElmer).

For synaptic sampling, GAD‐65/67 and SY‐38 positive puncta on the periphery of dentate nucleus neurons were originally detected as separate objects using the ‘automatic threshold’ function of the software. Areas of colocalization between these two populations were then defined as GABAergic synapses using the ‘exclusively combine’ command, creating a third population. Mitochondrial ‘objects’ were identified separately using the COX4 channel as a reference, while the synaptic mitochondrial population deviated after ‘compartmentalizing’ mitochondrial objects between GABAergic synapses. For Purkinje cell sampling, regions of interest (ROIs) were manually drawn and included the neuronal cell bodies. Mitochondrial ‘objects’ were recognized within each ROI, and protein intensity measures were taken from these. Detailed information on the development of each protocol is summarized in Figures 1 and 2. As image capture was performed on all x‐, y‐ and z‐planes, 3D reconstruction of synapses was possible, and this allowed synaptic numbers and size (volume) to be determined.

### Statistical analysis

For each area of interest (Purkinje cell body or GABAergic presynaptic terminal), the mean optical density (OD) of NDUFA13 and COX4 immunofluorescence was measured (Volocity®, UK, PerkinElmer, UK) and background corrected. The background‐corrected OD values were log transformed to normalize the data (yielding COX4_T_ and NDUFA13_T_). The mean and standard error for distribution of COX4_T_ were estimated, as well as the regression parameters and the standard error of the estimate for the regression of NDUFA13_T_ against COX4_T._ For each area of interest, the z score for the COX4 level was calculated, and the z score for NDUFA13 (NDUFA13_Z_) was calculated using the linear regression based on the level of COX4. Finally, Purkinje cells and/or GABAergic presynaptic terminals were classified based on standard deviation limits (for NDUFA13: normal if −2 < z < 2SD, low if z <−2SD, deficient if z <−3SD and very deficient if z <−4SD).

Once the z scores were derived, two‐sample *t* (for parametric data) and Mann–Whitney *U* tests (for nonparametric data) were performed to examine the differences in NDUFA13 and COX4 protein expression between different neuronal sub‐compartments or patients with different genetic defects. For testing paired nonparametric data, the Wilcoxon signed‐rank test was employed. Age, fixation time and PMI differences between patients and controls were tested using nonparametric Mann–Whitney *U* test. Spearman's correlation coefficient was used to test associations between continuous variables.

Statistical analyses were carried out using sas 9.3 (Cary, NC, USA) and Minitab® (16.1.0, UK).

We have not used Bonferroni or other adjustments for multiple testing in our analyses; where we are testing a priori hypotheses, this is appropriate [27]. However, the potential for more frequent type I statistical errors must be considered in interpretation of the *P* values resulting from these analyses.

### Laser microdissection, DNA extraction and pyrosequencing for mtDNA heteroplasmy in Purkinje cells and synapses

Sections of 15 μm‐thick frozen cerebellum mounted onto SuperFrost slides were subjected to double immunofluorescence for laser microdissection. The sections were typically left to air dry for 1.5 h and were fixed with 4% paraformaldehyde for 10 min at RT. Following this, sections were washed with Tris‐buffered saline (TBS) (two times × 2 min and one time × 10 min), blocked with 10% NGS for 30 min at RT and incubated in primary antibody solution overnight at 4^o^C. Primary antibodies included anti‐SY‐38 and anti‐GAD‐65/67 (Table S2). Sections were then washed with 10 mM TBS (three times × 5 min) and subjected to a biotinylated secondary antibody incubation (Biotin‐XX goat anti‐mouse IgG1) (A10519, Life Technologies, UK) for 30 min at RT. A secondary anti‐goat antibody conjugated with Alexa Fluor 488 (Life Technologies, UK) and a streptavidin‐conjugated Rhodamine Red‐X antibody (Jackson ImmunoResearch laboratories West Grove, PA, USA) were applied for 2 h at 4^o^C enabling double immunofluorescent labelling. Tissue auto‐fluorescence was blocked by incubating with 3% Sudan Black for 3 min.

Purkinje cells were identified using the 488 nm channel (fluorophore coupled to anti‐GAD‐65/67 antibody) and isolated at × 40 magnification using the ‘close‐cut and AutoLPC’ function on a PALM MicroBeam Laser microdissection microscope (Zeiss microscopy, UK). Inhibitory synapses to the dentate nucleus were recognized under the × 63 magnification as regions where both GAD‐65/67 and synaptophysin were localized and isolated using the ‘centre RoboLPC’ function. Laser dissected tissue was then subjected to overnight lysis using 10 μl of a DNA lysis buffer prior to PCR [28].

Extracted DNA was subjected to two rounds of PCR amplification using a biotinylated forward and reverse primer (Integrated DNA Technologies, Glasgow, UK) that would span the site of mutation (Table S3). Pyrosequencing was performed according to the manufacturer's instructions using a Pyromark Q24 platform (Qiagen, UK) and a mutation‐specific sequencing primer. The Pyromark Q24 software was employed to define mtDNA heteroplasmic levels by comparing the peak heights of mutant, wild‐type and known‐heteroplasmy mtDNA at the site of interest [29].

## Results

### Measuring respiratory chain protein expression using quadruple immunofluorescence

A subset of patients have been investigated previously using COX/SDH immunohistochemistry and other conventional immunohistochemical methods which relied upon the visualization of proteins using chromogens [9]. In this study, we have used a quantitative quadruple immunofluorescence technique in order to measure respiratory chain protein expression both in cell bodies and synaptic terminals. A similar approach to determine mitochondrial respiratory chain protein expression has recently been described in substantia nigra neurons and confirms the accuracy and reproducibility of the assay [30]. The current assay uses antibodies which are fluorescently labelled to allow visualization and quantification of COX4, NDUFA13, SY‐38 and GAD‐65/67 protein expression to look at mitochondrial mass, complex I, synaptophysin, and GABAergic cell bodies (Figure 1) and nerve terminals (Figure 2) simultaneously.

### Complex I deficiency detected in Purkinje cells

**Figure 3:**
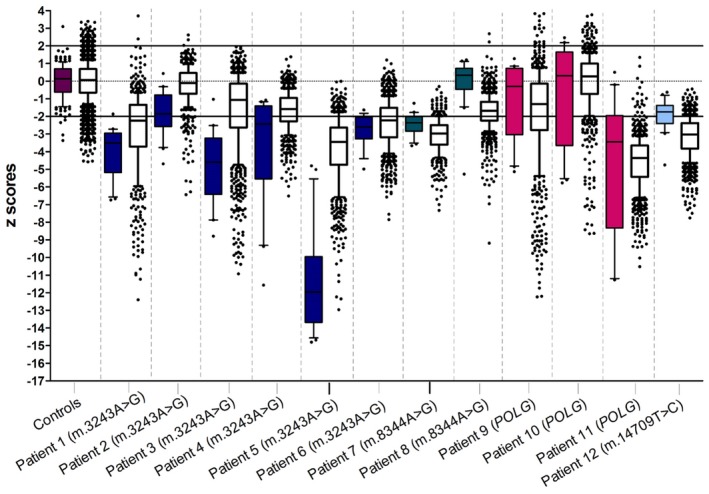
NDUFA13 protein expression in Purkinje cells and their inhibitory synapses on the dentate nucleus. Complex I deficiency in patient cerebellar Purkinje cells and inhibitory synapses is expressed as z score values. Coloured box plots represent Purkinje cell z values, and clear boxplots reflect inhibitory synapse z values. In Purkinje cells, the most severe deficiency is generally observed in patients with the m.3243A > G point mutation (Patients 1–6), with Patient 5 (m.3243A > G) showing the most dramatic loss of NDUFA13 protein expression (Median z score = −12). Two individuals with recessive POLG mutations (Patient 9 and 10) demonstrate relatively high NDUFA13 protein expression. Patients with the same genetic defect show variability in their complex I expression (Patient 2 *vs.* Patients 1 and 3 for m.3243A > G; Patient 7 *vs.* Patient 8 for m.8344A > G). In inhibitory synapses, the majority of patients revealed variably decreased complex I expression, with Patients 5 (m.3243A > G) and 11 (POLG) possessing the most deficient synaptic terminals, while Patients 2 (m.3243A > G) and 10 (POLG) are not different from controls.

Purkinje cell bodies were identified by diffuse staining of GAD‐65/67 throughout the cell body (Figure 1). In control neurons, mitochondria (as judged by COX4 expression) were abundant and showed co‐localization with complex I subunit (NDUFA13). There was no difference in COX4 protein expression between patient and control Purkinje cells (two‐sample *t* test, *P* = 0.323) in agreement with previous studies that rarely report deficiency of this protein [9]. However, in some Purkinje cells from patients, there was loss of NDUFA13, indicating complex I deficiency (Figure 1), while in Basket cells, NDUFA13 appeared intact. Quantification of protein expression confirmed complex I deficiency and enabled comparison between patient and control neurons in order to explore the severity of the defect. Complex I deficiency (z <−3SDs) was observed in patients carrying the m.3243A > G mutation (Patients 1, 3 and 5) and a patient carrying autosomal recessive *POLG* mutations (Patient 11), while decreased complex I levels (z <−2SDs) were observed in patients with m.3243A > G (Patient 4 and 6) and m.8344A > G (Patient 7) point mutations (Figure 3). Our data showed Purkinje cell deficiency is more severe in patients with the m.3243A > G point mutation compared with the level of deficiency in the other patients when genetic defects were grouped (m.8344A > G, m.14709T > G and *POLG)* (Mann–Whitney *U* test, *P* = 0.0306). There was considerable variation in the extent of deficiency between patients with the same genetic defect as has previously been reported [9]. Spearman's rank correlation analysis showed that PMI (Spearman's rank rho = −0.242, *P* = 0.449) and fixation time (rho = −0.035, *P* = 0.913) did not affect the derived z score values for complex I deficiency in Purkinje cells.

### Complex I deficiency in GABAergic presynaptic terminals

**Figure 4:**
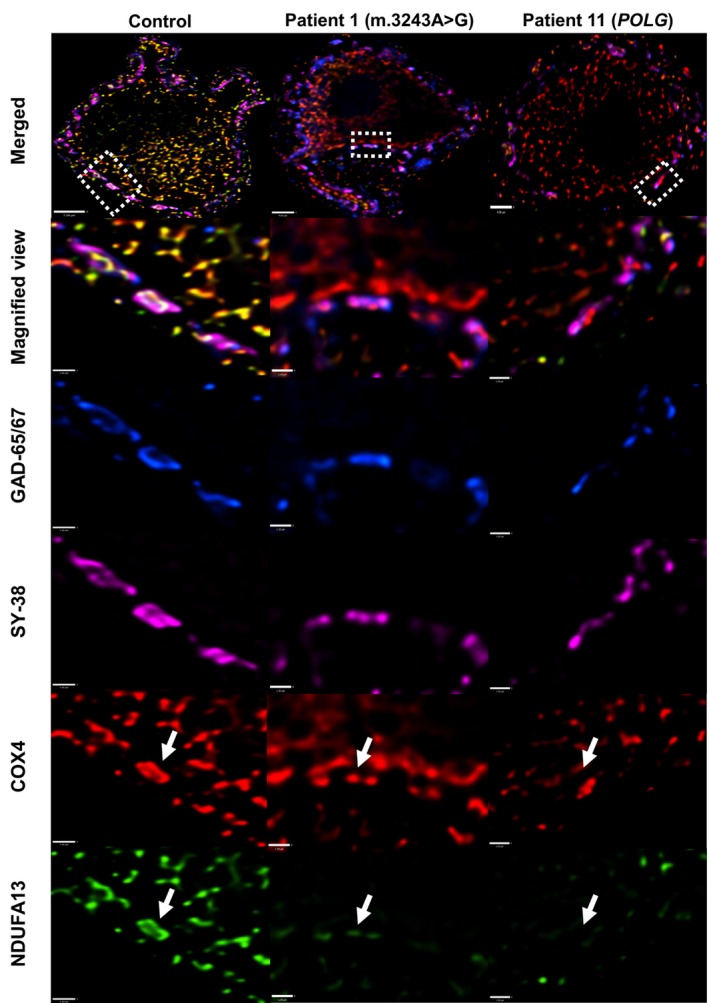
Complex I deficiency in inhibitory synapses on dentate nucleus neurons. Quantification of NDUFA13 protein expression in GABAergic synapses around dentate nucleus neurons (top panel; scale bar: 7 μm). Control inhibitory synapses have equal expression of COX4 (mitochondrial mass) and NDUFA13 (white arrows – left panel), indicating intact complex I expression. In contrast, mitochondria in patient inhibitory presynaptic terminals have decreased complex I expression (white arrows), indicating mitochondrial dysfunction in these synapses. There is a severe reduction in complex I expression relative to mitochondria (Patient 5; m.3243A > G), while in other patients, there is only a small difference in complex I to COX4 expression (Patient 11; POLG). Scale bar: 1.5 μm.

Evaluation of Purkinje cell synapses on dentate nucleus neurons revealed matched expression of NDUFA13 and COX4 in control tissue. In patient tissue, it was clear that some synapses had low expression of NDUFA13 (Figure 4). Protein intensity quantification confirmed no difference in COX4 expression in control and patient synapses (two‐sample *t* test, *P* = 0.620); however, NDUFA13 relative to COX4 intensity levels confirmed decreased expression and low z scores compatible with complex I deficiency (z <−3SDs) (Figure 3; Table 2). This respiratory chain deficiency is variable, with patient synapses demonstrating either a minor (Patient 1; m.3243A > G mutation) or a severe (Patient 11, recessive *POLG* mutations) decrease in NDUFA13 expression despite maintained COX4 expression (Figure 4). Similar to Purkinje cells, synaptic NDUFA13 deficiency was seen in patients with different genetic defects, but there was considerable variability in the extent of deficiency even in patients with the same genetic defect. Complex I deficiency in inhibitory presynaptic terminals was not affected by either PMI (rho = −0.126, *P* = 0.696) or fixation time (rho = −0.007, *P* = 0.983) (Spearman's rank correlation).

**Table 2:** Percentage complex I deficiency in Purkinje cells and their GABAergic axonal terminals

	Controls (%)	Pt1 (%)	Pt2 (%)	Pt3 (%)	Pt4 (%)	Pt5 (%)	Pt6 (%)	Pt7 (%)	Pt8 (%)	Pt9 (%)	Pt10 (%)	Pt11 (%)	Pt12 (%)
Purkinje cells													
Normal (−2 < CI_z < 2)	98.73	5	55	5	45	0	21.05	25	95	70	75	26.32%	61.90
Low (CI_z <−2)	1.27	20	30	15	15	0	42.11	60	0	5	0	15.79%	33.33
Deficient (CI_z <−3)	0	35	10	20	0	0	21.05	15	0	5	0	21.05%	0
Very deficient (CI_z <−4)	0	40	5	60	40	100	15.79	0	5	20	25	36.84%	4.76
GABAergic presynaptic terminals													
Normal (−2 < CI_z < 2)	97.13	43.01	92.45	68.28	66.96	13.62	41.60	10.08	65.68	64.67	90.20	2.32%	12.02
Low (CI_z <−2)	2.32	23.89	5.25	11.38	22.36	22.83	29.77	41.53	26.91	12.75	5.43	5.96%	37.40
Deficient (CI_z <−3)	0.37	10.80	0.82	5.41	7.24	26.57	17.75	33.47	5.35	5.84	1.30	30.24%	30.13
Very deficient (CI_z <−4)	0.18	22.30	1.48	14.93	3.43	36.98	10.89	14.92	2.06	16.73	3.07	61.48%	20.45

The percentage of Purkinje cells and inhibitory presynaptic terminals that are normal (−2 < CI_z < 2), low (CI_z <−2), deficient (CI_z <−3) or very deficient (C1_z <−4) for NDUFA13 protein expression in controls and patients with mitochondrial disease. Controls have very little (if any) deficiency, while patient cells and/or synapses are variably deficient.

**Figure 5:**
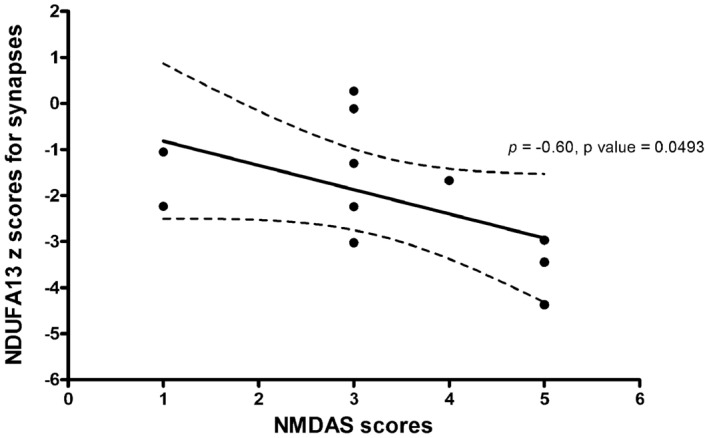
Correlation between NMDAS score for cerebellar ataxia and NDUFA13 protein expression in inhibitory presynaptic terminals. More severe ataxia scores are related to lower z scores for NDUFA13 expression (Spearman's rho = −0.60, *P* value = 0.0493).

Analysis of NMDAS scores and NDUFA13 expression in synapses reveals a statistically significant relationship between higher NMDAS scores, indicating a more severe ataxia, and lower z scores, indicating more complex I deficiency (Spearman's rho = −0.60, *P* value = 0.0493) (Figure 5).

### Comparison of complex I deficiency in Purkinje cells and in GABAergic presynaptic terminals

For both Purkinje cells and their synapses, there is considerable variation between patients in the degree of deficiency observed. While patients harbouring the m.3243A > G showed significantly more complex I deficiency in Purkinje cells than the other patients when these were grouped, this was not true for the level of deficiency in synapses, which was comparable in all patients (Figure 3). Interestingly, while all patients with the m.3243A > G mutation showed more deficiency (lower median z scores) in Purkinje cell bodies than synapses, all patients in the group of other mutations (m.8344A > G, m.14709T > C and *POLG* mutations) showed the reverse trend. The difference between these two groups was statistically significant (Mann–Whitney *U* test, *P* = 0.0051), though this was not an *a priori* hypothesis and the *P* value needs to be interpreted with caution. There is no difference in complex I expression levels between Purkinje cell bodies and GABAergic synapses in the group of patients as a whole (Wilcoxon signed‐rank test of differences between z scores in Purkinje cells and synapses, *P* = 0.666).

### GABAergic synaptic loss and remodelling

**Figure 6:**
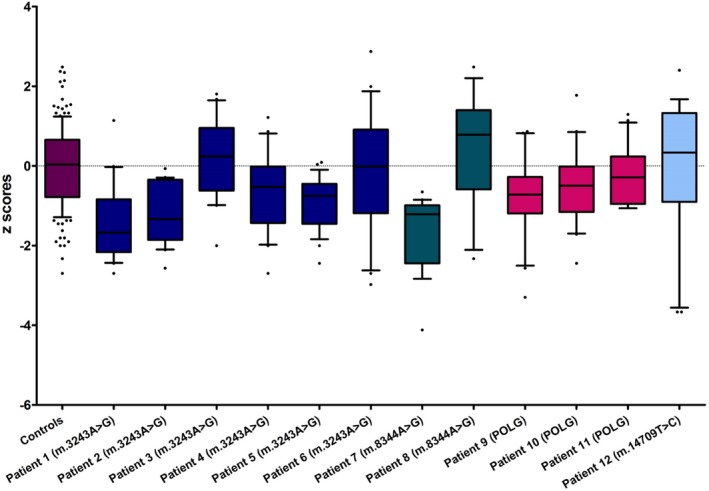
Changes in the number of inhibitory presynaptic terminals. The number of GABAergic synapses counted on each dentate nucleus neuron (*n* = 20) expressed as z score values. Decreased numbers of inhibitory presynaptic terminals is detected in eight out of 12 patients. All patients with recessive POLG mutations exhibit synapse loss, in addition to the majority of patients harbouring the m.3243A > G point mutation. Intriguingly, Patients 3 (m.3243A > G), 8 (m.8344A > G) and 12 (m.14709T > C) have increased numbers per cell compared with controls.

The majority of patients (Patients 1, 2, 4, 5, 7, 9–11) revealed a decrease in the number of GABAergic presynaptic terminals on dentate nucleus neurons relative to controls (Figure 6) though this decrease lies within the ±2 SDs limits. This suggests synaptic loss in conjunction with Purkinje cell loss (Table 1), and indeed, there is a correlation between Purkinje cell loss and the loss of inhibitory synapses on dentate nucleus neurons (Spearman's rank correlation coefficient = −0.74; *P* value = 0.02). Patients 3, 8 and 12 do not demonstrate synaptic loss, which can be explained by preservation of Purkinje cells (Table 1). Interestingly, there is also a correlation between the number and the size (volume) of GABAergic synapses in patients with an increase in volume of residual inhibitory synapses when there is synaptic loss (Spearman's rank correlation coefficient = −0.615; *P* value = 0.037). Synaptic numbers and volume were not affected by PMI (rho = 0.143, *P* = 0.736 for synaptic numbers and rho = 0.595, *P* = 0.120 for synaptic volume) or by fixation length (rho = 0.011, *P* = 0.980 for synaptic numbers and rho = −0.688, *P* = 0.059 for synaptic volume) (Spearman's rank correlation).

### MtDNA heteroplasmy in Purkinje cell bodies *vs.* inhibitory presynaptic terminals

**Figure 7:**
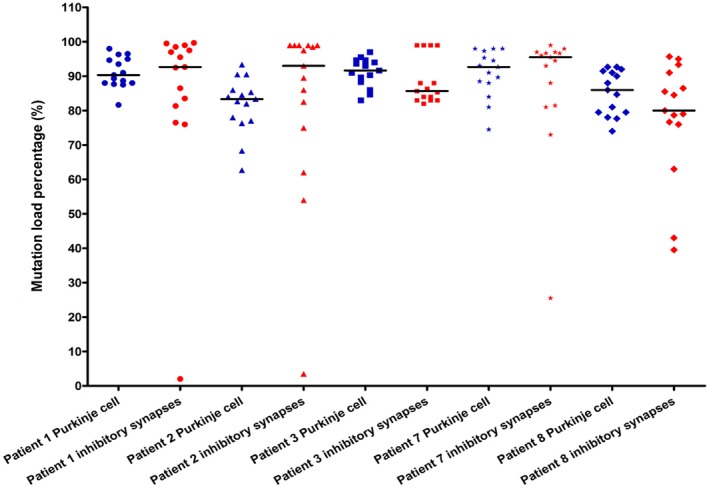
MtDNA mutation load in isolated Purkinje cells and inhibitory presynaptic terminals. Heteroplasmy levels for individually isolated Purkinje cell bodies and inhibitory presynaptic terminals from five patients harbouring mtDNA point mutations (m.3243A > G and m.8344A > G). Individual symbols represent independent read‐outs, and bars represent medians (*n* = 15 for both populations).

Assessment of mutated mtDNA heteroplasmy for m.3243A > G and m.8344A > G in both Purkinje cells and inhibitory synapses did not show any significant difference in the levels of mutated mtDNA between the two neuronal sub‐compartments (Wilcoxon signed‐rank test of differences between mutation load percentage in Purkinje cells and synapses, *P* = 1) (Figure 7).

## Discussion

Cerebellar dysfunction due to mitochondrial impairments is increasingly recognized as an important cause of ataxia. Ataxia is common and an important cause of disability in patients with mitochondrial disease [8, 31, 32]. Neuropathological studies provide important clues into disease mechanisms and reveal that Purkinje cells are particularly vulnerable to degeneration and cell death. In this study, we aimed to clarify the impact of mitochondrial defects on the microcircuitry of the cerebellum by focussing on the GABAergic Purkinje cells and their synapses. To achieve this, we have used a quadruple immunofluorescent technique to detect complex I within mitochondria in GABAergic synapses from 12 patients. We show that complex I deficiency is present in both Purkinje cell bodies and their inhibitory synapses, with no overall difference in NDUFA13 expression between the two regions. There was loss of synapses in some patients, and this was associated with enlarged remaining presynaptic terminals. Finally, in those patients with heteroplasmic mtDNA point mutations, we observed no difference in mtDNA mutation level between Purkinje cell bodies and their synapses.

Previous neuropathological studies of patients with mitochondrial disease have shown a positive relationship between neuronal cell loss and high respiratory chain deficiency in remaining cells [9]. In the majority of previous studies, mitochondrial protein changes are assessed using conventional histochemical (sequential COX/SDH reaction) and immunohistochemical methods. These are limited because they are subjective and only enable the reliable detection of cells with either complete loss of enzyme/protein expression or high enzyme/protein levels without correcting for mitochondrial mass. Hence, any mild or intermediate changes in respiratory chain protein expression may be overlooked. We recognized the need to use an accurate and reliable method to document respiratory chain deficiency and therefore employed quadruple immunofluorescence in order to precisely and reliably quantify respiratory chain protein expression within specific cell types and their sub‐cellular domains. We quantified complex I deficiencies in Purkinje cells and their GABAergic presynaptic terminals through the use of four fluorescently labelled protein markers, labelling mitochondria, complex I, GABA‐expressing neurons and synapses. Previous studies have shown that the NDUFA13 subunit plays an essential role in the assembly and activity of complex I, and indeed a recent study reports NDUFA13 mutations which lead to complex I instability [33]. Due to a lack of available histochemical assays to measure complex I activity at the single cell level in tissues, however, we cannot measure complex I activity biochemically. We infer that NDUFA13 expression with a z score of −3 or less suggests complex I deficiency which is likely to correlate with loss of complex I activity.

We show that Purkinje cell bodies and their synaptic terminals have decreased protein expression in patients relative to controls. This is a significant observation for Purkinje cells from patients with the m.3243A > G mutation where prominent complex I deficiency was observed. This is consistent with previous studies in skeletal muscle from patients with m.3243A > G and m.8344A > G mutations where low complex I activity and low expression of complex I subunits are a common finding [34, 35, 36]. NDUFA13 protein expression is decreased in patients with different genetic defects, and protein deficiency is seen in both Purkinje cell bodies and inhibitory presynaptic terminals. Overall, the degree of complex I expression is similar between the two neuronal domains. It is interesting that NDUFA13 deficiency in synapses correlates well with ataxia which suggests that synaptic pathology could contribute to mitochondrial disease pathogenesis in these patients.

In addition, for those patients with heteroplasmic mtDNA mutations, molecular genetic investigation shows that the levels of mutated compared with wild‐type mtDNA are similar between Purkinje cell bodies and their inhibitory synapses which are consistent with the equivalent level of NDUFA13 deficiency in both compartments.

Damaged mitochondria are recycled via a process known as mitophagy that takes place in the neuronal cell body [37]. Under normal conditions, dysfunctional synaptic mitochondria are retrogradely transported back to the cell body to undergo recycling, leaving the synapse with healthy organelles that would be able to provide the region with energy [38]. Comparable levels of NDUFA13 expression and heteroplasmic mtDNA between Purkinje cell bodies and GABAergic synapses in patients were observed, and we are uncertain why the deficient mitochondria were not retrogradely transported for degradation.

Mitochondrial function is important for sustaining activity‐dependent energy requirements in the presynaptic terminal [39]. Mitochondrial‐generated ATP is required for efficient neurotransmission by fuelling synaptic vesicle docking to the presynaptic membrane and neurotransmitter release and uptake to/from the synaptic cleft [40, 41]. Moreover, complex I activity has been demonstrated to be a critical factor for synaptic oxygen consumption [17, 18], while regulation of the mitochondrial calcium buffering capacity is important for recovery following repetitive depolarization [7]. It is therefore likely that presynaptic complex I deficiency would be detrimental for normal synaptic activity.

Reconstruction of GABAergic presynaptic terminals allowed us to evaluate synaptic numbers and size. The number of inhibitory contacts detected on dentate nucleus neurons is decreased in the majority of patients, while synaptic volume is increased. We speculate that this could be a compensatory mechanism in an attempt to maintain contact area by adjusting synaptic volume in case of contact loss. This would be in agreement with previous ultrastructural studies in the frontal cortex of patients with Alzheimer's disease where a strong negative correlation was found between synaptic contact size and density [42].

Post‐mortem studies in common neurodegenerative diseases including Alzheimer's, Huntington's and Parkinson's have suggested that loss of synapses occurs as an early event, prior to loss of the neuronal cell body, a process known as ‘dying‐back’ [43, 44]. Investigating whether this is also applicable in the case of mitochondrial disease is interesting but also challenging as our study is performed on post‐mortem human brains and represents end‐stage disease. The correlation between synaptic contact numbers and the percentage of Purkinje cell loss suggests that the documented changes on synapse numbers might simply be a reflection of Purkinje cell loss. Further studies using an animal model would provide greater insights into temporal evolution of neurodegeneration and would be a useful approach to understand whether synaptic abnormalities precede neuronal cell loss.

## Conclusion

In this study, we have quantified mitochondrial respiratory chain complex I expression in Purkinje cells and their inhibitory projections and provided quantitative evidence of morphological abnormalities in synapses in patients with mitochondrial disease and ataxia. Our data suggest that patients harbouring the m.3243A > G mutation show the highest levels of complex I deficiency in Purkinje cells. However, in general, there is no difference in the level of deficiency between the two neuronal sub‐compartments, and in those patients with heteroplasmic mtDNA mutations, there are similar levels of mutated mtDNA. We report fewer GABAergic synapses and enlarged residual synapses contacting the dentate nucleus, which could suggest altered cerebellar circuitry that might contribute to ataxia seen in these patients. Further work is crucial to dissect out the impact of complex I deficiency on neurons and their synapses in a functional system to understand the mechanisms of degeneration in mitochondrial disease.

## Author contributions

NZL, DMT and RWT contributed considerably to the perception of the study. AC, NZL, DMT and AL designed the study. DMT acquired the clinical information. AC acquired the data. AC and JPG performed data analysis. AC, JPG, NZL and DMT contributed to data interpretation, and all authors contributed to the critical revision of and gave their approval to the manuscript.

## Conflict of Interest

The authors declare there are no competing financial interests.

## Supporting information

Figure S1. The effect of Sudan Black treatment.
Table S1. Details of control brain tissue used in the study.
Table S2. The characteristics of primary and secondary antibodies used in the study.
Table S3. Primer sequences used in the study.
