# Supplementary material for: Investigating complex I deficiency in Purkinje cells and synapses in patients with mitochondrial disease
Source: Neuropathol Appl Neurobiol. 2015 Sep 30;42(5):477–92. doi: 10.1111/nan.12282 (PMC4973693; doi:10.1111/nan.12282)
Supplement: Supplementary file 1 — Figure S1. The effect of Sudan Black treatment. Table S1. Details of control brain tissue used in the study. Table S2. The characteristics of primary and secondary antibodies used in the study. Table S3. Primer sequences used in the study. [file NAN-42-477-s001.docx]

**Supplementary Data**

**Supplementary Table 1: Details of control brain tissue used in the study**

| Type | Age at death (years) | Sex | Length of fixation (weeks) | Post-mortem delay (hours) | Cause of death |
| --- | --- | --- | --- | --- | --- |
| Control | 78 | Female | 5 | 23 | metastatic oesophageal carcinoma |
| Control | 69 | Female | 4 | 67 | Gastric cancer |
| Control | 74 | Female | 12 | 16 | Lung cancer |
| Control | 78 | Female | 8 | 34 | Metastatic cancer - primary origin unknown (probably ovarian) |
| Control | 48 | Male | 1 | 72 | Coronary artery atherosclerosis |
| Control | 48 | Male | 1 | 46 | Coronary artery atherosclerosis |
| Control | 61 | Male | 1 | 61 | ND^^[[1]](#footnote-1)^^ |
| Control | 48 | Male | 1 | 43 | Coronary artery thromvosis and atherosclerosis |
| Control | 25 | Male | 1 | 53 | ND |
| Control | 44 | Male | 1 | 83 | Complications of bronchopneumonia and coronary artery atherosclerosis |

Information on control tissue obtained from the NBTR and the MRC Sudden Death Brain and Tissue Bank, Edinburgh.


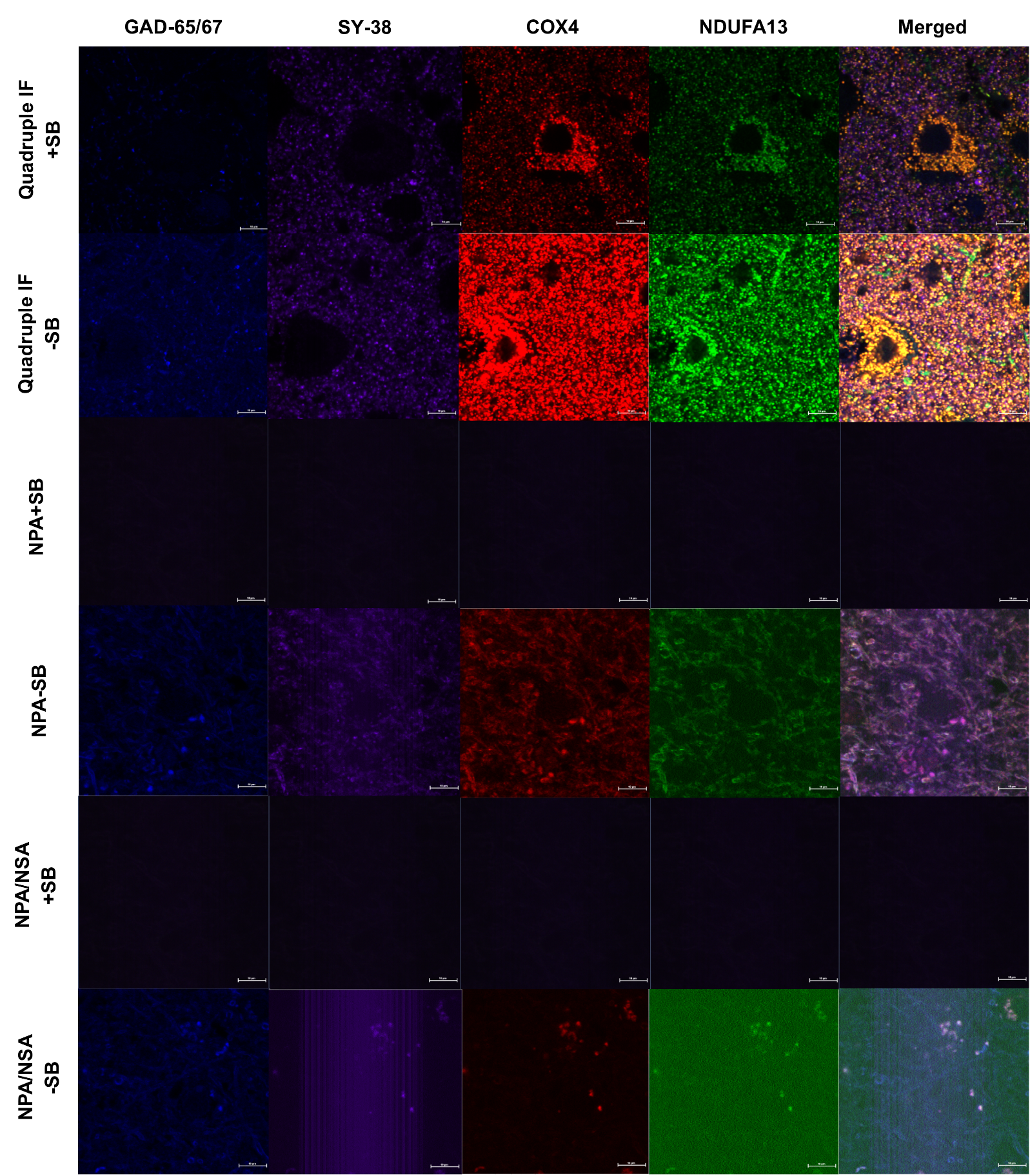


**Supplementary Figure 1: The effect of Sudan Black treatment**

The importance of Sudan black b incubation in increasing the signal-to-noise ration and in removing the background. Scale bar: 10µm. SB –Sudan black, NPA – no primary antibodies, NSA – no secondary antibodies

**Supplementary Table 2: The characteristics of primary and secondary antibodies used in the study**

| Primary antibody | Epitope | Host | Source | Catalog No. | Antigen retrieval | Optimal dilution | Optimal incubation |
| --- | --- | --- | --- | --- | --- | --- | --- |
| Synaptophysin (SY-38) | presynaptic vesicles | Mouse monoclonal - IgG1 | DAKO | M0776 | EDTA (1mM); 2100 antigen retriever | 1 in 50 | 4°C overnight |
| Glutamic acid decarboxylase 65/67 (GAD-65/67) | GABAergic neurons and Inhibitory axonal terminals | Rabbit polyclonal - IgG | Sigma-Aldrich | G5163 | EDTA (1mM); 2100 antigen retriever | 1 in 800 | 4°C overnight |
| NDUFA13 | NDUFA13 subunit | Mouse monoclonal - IgG2b | Abcam | ab110240 | EDTA (1mM); 2100 antigen retriever | 1 in 100 | 4°C overnight |
| COX4 | COX4+COX4L2 | Mouse monoclonal - IgG2a | Abcam | ab110261 | EDTA (1mM); 2100 antigen retriever | 1 in 200 | 4°C overnight |
| **Secondary antibody** | **Epitope** | **Host** | **Source** | **Catalog No.** | **Antigen retrieval** | **Optimal dilution** | **Optimal incubation** |
| Biotin-XX | IgG1 | Goat | Life Technologies | A10519 | N/A | 1 in 100 | 30 minutes, RT^^[[2]](#footnote-2)^^ |
| Streptavidin, Alexa Fluor® 647 Conjugate | Biotin | Goat | Life Technologies | S32357 | N/A | 1 in 100 | 30 minutes, RT |
| Alexa Fluor® 405 | Rabbit IgG (H+L) | Goat | Life Technologies | A31556 | N/A | 1 in 100 | 30 minutes, RT |
| Alexa Fluor® 488 | Mouse IgG2b (γ2b) | Goat | Life Technologies | A2114 | N/A | 1 in 100 | 1 hour, RT |
| Alexa Fluor® 546 | Mouse IgG2a (γ2a) | Goat | Life Technologies | A21133 | N/A | 1 in 100 | 30 minutes, RT |
| Alexa Fluor® 488 | Anti-Rabbit IgG (H+L) | Goat | Life Technologies | A11008 | N/A | 1 in 100 | 2 hours, 4°C |
| Rhodamine Red™-X (RRX) Streptavidin | Biotin | Goat | Jackson ImmunoResearch | 016-290-084 | N/A | 1 in 100 | 2 hours, 4°C |

The antibodies employed for quadruple immunofluorescence on FFPE tissue and double immunofluorescence on frozen tissue and their optimized working conditions. RT – room temperature.

|  | Mitochondrial DNA sequence (NC_012920.1) | Tm( ͦ C) |
| --- | --- | --- |
| **m.3243A>G primer sequences** | | |
| 5'-biotin forward primer (nt3143-3163) | 5'-TAAGGCCTACTTCACAAAGCG | 62 |
| Reverse primer (nt3331-3353) | 5'- GCGATTAGAATGGGTACAATGAG |  |
| Sequencing primer (nt3244-3258) | 5'- ATGCGATTACCGGGC |  |
| **m.8344A>G primer sequences** | | |
| 5'-biotin forward primer (nt8240-8264) | 5'-TTT GAA ATA GGG CCC GTA TTT ACC | 62 |
| Reverse primer (nt8363-8387) | 5'-CGG TAG TAT TTA GTT GGG GCA TTT |  |
| Sequencing primer (nt8347-8367) | 5'-ATT TCA CTG TAA AGA GGT GT |  |

**Supplementary Table 3: Primer sequences used in the study**

The mtDNA sequences for the primers used to define the heteroplasmic mtDNA level.

1. ND – Not Determined [↑](#footnote-ref-1)
2. RT – Room Temperature [↑](#footnote-ref-2)
